# Natural variation in chemosensation: lessons from an island nematode

**DOI:** 10.1002/ece3.902

**Published:** 2013-11-28

**Authors:** Angela McGaughran, Katy Morgan, Ralf J Sommer

**Affiliations:** Department for Evolutionary Biology, Max Planck Institute for Developmental BiologyTübingen, D-72076, Germany

**Keywords:** Chemoattraction, ecology, La Réunion Island, natural variation, nematode, *Pristionchus pacificus*.

## Abstract

All organisms must interact with their environment, responding in behavioral, chemical, and other ways to various stimuli throughout their life cycles. Characterizing traits that directly represent an organism's ability to sense and react to their environment provides useful insight into the evolution of life-history strategies. One such trait for the nematode *Pristionchus pacificus*, chemosensation, is involved in navigation to beetle hosts. Essential for the survival of the nematode, chemosensory behavior may be subject to variation as nematodes discriminate among chemical cues to complete their life cycle. We examine this hypothesis using natural isolates of *P. pacificus* from La Réunion Island. We select strains from a variety of La Réunion beetle hosts and geographic locations and examine their chemoattraction response toward organic compounds, beetle washes, and live beetles. We find that nematodes show significant differences in their response to various chemicals and are able to chemotax to live beetles in a novel assay. Further, strains can discriminate among different cues, showing more similar responses toward beetle washes than to organic compounds in cluster analyses. However, we find that variance in chemoattraction response is not significantly associated with temperature, location, or beetle host. Rather, strains show a more concerted response toward compounds they most likely directly encounter in the wild. We suggest that divergence in odor-guided behavior in *P. pacificus* may therefore have an important ecological component.

## Introduction

Understanding the ways in which organisms interact with their surroundings requires identification and characterization of the phenotypic traits involved in environmental response. Ecologically relevant traits, such as those involved in environmental sensing and monitoring, are particularly useful, because they represent an organism's ability to identify and cope with environmental change. In nematodes, an important component of the sensing mechanisms mediating interactions with external stimuli is olfactory-based (Bargmann et al. 1993; Jovelin et al. 2003; Hallem et al. 2011). Through odor detection, nematodes have been shown to navigate toward to or away from host excretory products (Grewal et al. 1993), bacterial symbionts and changes in pH (Pye and Burman 1981), temperature (Burman and Pye 1980), electrical field (Shapiro-Ilan et al. 2012), and various plant volatile compounds (Laznik and Trdan 2013).

The hermaphroditic nematode *Pristionchus pacificus* has an additional environmental interaction that utilizes its olfactory apparatus – a necromenic association with host beetles (Herrmann et al. 2006, 2011; Hong and Sommer 2006). This association begins as *P. pacificus* selectively follows remote volatile cues from a potential host. Upon contact and recognition, the nematode passively inhabits the host in an arrested “dauer” stage until the beetles' demise (Weller et al. 2010). Thereafter, *P. pacificus* detects a suite of chemical substances that cause it to resume development, feeding on the microbes that flourish on the beetle carcass (Herrmann et al. 2006, 2007; Mayer and Sommer 2011).

**Figure 1:**
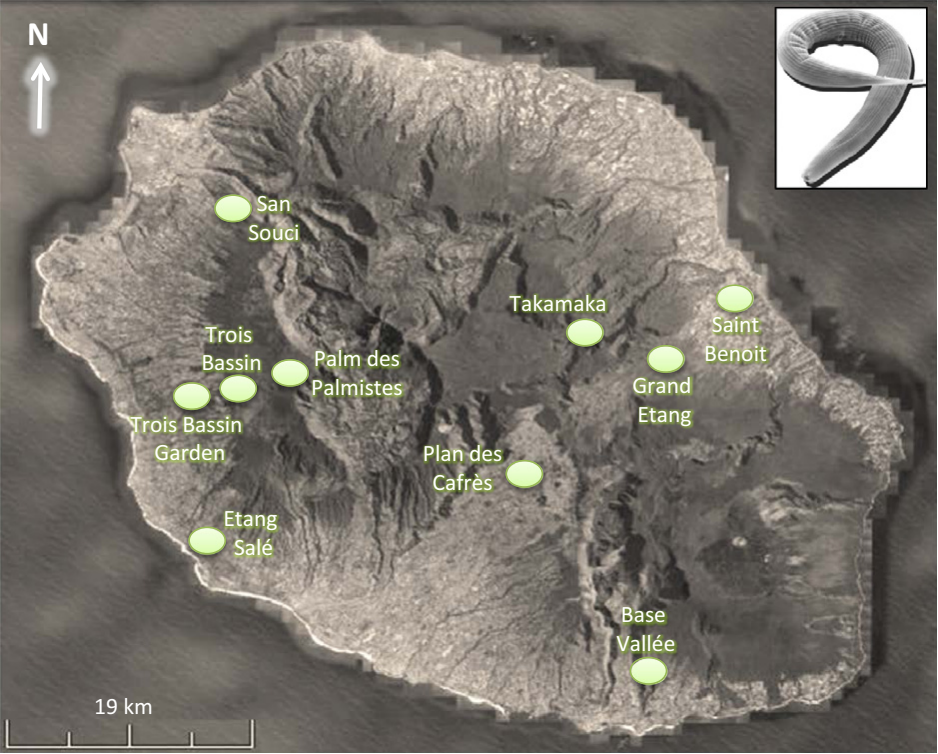
Map of La Réunion Island to show the complex topography and the approximate collection locations of *Pristionchus pacificus* strains used in the current study (see Table 1 for further information); Inset, top right: The nematode *P. pacificus*.

*Pristionchus pacificus* has been found in association with multiple scarab beetle species throughout a cosmopolitan distribution that encompasses Africa, Asia, Europe, America, and the Mascareigne Islands of the Indian Ocean (Herrmann et al. 2006, 2011). In the latter location, La Réunion Island (2–3 Ma; 2512 km^2^; Fig. 1) appears to be an oasis for *P. pacificus* (e.g., Herrmann et al. 2011; Morgan et al. 2012; McGaughran et al. 2013). Considered to be a major biodiversity hotspot (Myers et al. 2000; Thebaud et al. 2009), La Réunion is the largest and most complex island in the Mascareigne group, both topographically and ecologically. With a mix of inland steep relief and coastal lowlands, the island is home to a complex of habitat types or “ecozones”, within which species diversity and endemism broadly differ (Strasberg et al. 2005).

In recent years (Herrmann et al. 2011; Morgan et al. 2012), nematode samples have been collected across the Réunion ecozone tableau from several scarab beetle species, both recently introduced (<2000 ya; Vercambre et al. 1991; Cheke and Hume 2008) and endemic. Thus, ecozone (i.e., geological, climatic) and host differences among strains together provide a unique template from which examination of ecological trait differentiation in the context of host choice may be possible.

Chemosensory behavior has been extensively studied in the model nematode *Caenorhabditis elegans*, which has been shown to follow both water-soluble and volatile cues (e.g., Bargmann et al. 1993). In *Pristionchus* nematodes, closely related pairs of species show significantly diverged attraction to insect- and plant-related organic compounds in a species-specific manner (Hong and Sommer 2006; Rae et al. 2008), suggesting that the genus has evolved to intercept the chemical communication system of its members' insect hosts (Hong et al. 2008; Sokolowski and Fitzpatrick 2008). Intraspecific chemoattraction differentiation has also been demonstrated among globally distributed *P. pacificus* strains in their response to beetle pheromones (Hong and Sommer 2006; Hong et al. 2008), but has currently not been studied systematically utilizing the unique La Réunion system.

Here, we use chemoattraction assays to examine whether chemosensory behavior in *P. pacificus* may be subject to high degrees of variation as nematodes discriminate among chemical cues to complete their life cycle. To achieve this, we examine chemosensation in the context of both host beetle and habitat differences among strains. We demonstrate that La Réunion *P. pacificus* strains show extreme variation in their odor-guided behavior and that this variance has an ecological component that is driven by concerted responses among strains toward compounds most likely directly encountered in the wild.

## Materials and Methods

### Nematode strains

In total, 62 nematode strains were selected for transfer from laboratory culture to nematode growth medium (NGM) agar plates to start isogenic lines for various chemosensation assays. Of these, 61 strains were originally isolated from several locations/scarab beetle hosts across La Réunion Island (Fig. 1, Table 1). One further strain (RS2333) originally isolated from soil in Pasadena (California, USA) serves as a reference strain for our laboratory and was used for quality control in the current study. During the various experimental periods, all strains were maintained, following standard protocols, at 20°C on NGM-agar media and fed with *Escherichia coli* OP50 strain (Brenner 1974).

**Table 1:** List of *Pristionchus pacificus* strains used in this study, with their strain code, date of sampling, strain and host beetle origin, genetic lineage (Morgan et al. 2012), and the chemoattraction experiment they were used for

					Experiment			
					
Strain code	Date of sampling	Strain origin	Host origin	Genetic lineage	STD	TMP	MTP	LIV
RS2333	2010	California	Soil	A	*			
RS5334	2010	Trois Bassin	*Oryctes borbonicus*	C	*		*	
RS5336	2010	Trois Bassin	*O. borbonicus*	C	*		*	*
RS5337	2010	Trois Bassin	*O. borbonicus*	C	*			
RS5342	2010	Basse Vallée	*Adoretus* sp.	C			*	
RS5347	2010	Trois Bassin	*O. borbonicus*	C	*		*	*
RS5350	2010	Etang Salé	*Hoplia retusa*	C	*			
RS5351	2010	Trois Bassin	*O. borbonicus*	C	*		*	
RS5378	2010	Trois Bassin	*O. borbonicus*	C	*	*		
RS5385	2010	Trois Bassin	*O. borbonicus*	C	*			*
RS5397	2010	Trois Bassin	*O. borbonicus*	C	*	*		
RS5398	2010	Trois Bassin	*O. borbonicus*	C	*			
RS5399	2010	Trois Bassin	*O. borbonicus*	C	*		*	
RS5402	2010	Trois Bassin	*H. retusa*	C			*	*
RS5403	2010	Trois Bassin	*H. retusa*	C			*	
RS5404	2010	Trois Bassin	*H. retusa*	C				*
RS5405	2010	Trois Bassin	*H. retusa*	C	*		*	*
RS5406	2010	Basse Vallée	*Adoretus* sp.	D			*	
RS5409	2010	Grand Etang	*Adoretus* sp.	C	*		*	
RS5410	2010	Grand Etang	*Adoretus* sp.	C	*	*	*	
RS5411	2010	Grand Etang	*Adoretus* sp.	C	*	*	*	
RS5415	2010	Saint Benoit	*Maladera affinis*	C	*	*	*	
RS5418	2010	Saint Benoit	*M. affinis*	C	*	*	*	
RS5419	2010	Saint Benoit	*M. affinis*	A			*	*
RS5421	2010	Saint Benoit	*M. affinis*	A			*	
RS5423	2010	Saint Benoit	*M. affinis*	A			*	
RS5426	2010	Trois Bassin Garden	*Hoplochelus marginalis*	C				*
RS5427	2010	Trois Bassin Garden	*H. marginalis*	C	*			*
RS5429	2010	Trois Bassin Garden	*H. marginalis*	C	*			*
RS5430	2010	Trois Bassin Garden	*M. affinis*	C	*			*
RSA037	2011	Basse Vallée	*Adoretus* sp.	C			*	
RSA038	2011	Basse Vallée	*Adoretus* sp.	D			*	
RSA044	2011	Basse Vallée	*Adoretus* sp.	D			*	
RSA046	2011	Grand Etang	*Adoretus* sp.	C			*	
RSA059	2011	Grand Etang	*Adoretus* sp.	D			*	
RSA080	2011	Plan des Cafrès	*H. retusa*	C			*	
RSA085	2011	Plan des Cafrès	*H. retusa*	C			*	
RSA089	2011	Trois Bassin	*H. retusa*	C			*	
RSA091	2011	Trois Bassin	*H. retusa*	C			*	
RSA096	2011	San Souci	*O. borbonicus*	C			*	
RSA102	2011	San Souci	*O. borbonicus*	D			*	
RSA104	2011	San Souci	*O. borbonicus*	C			*	
RSA108	2011	San Souci	*O. borbonicus*	C			*	
RSA111	2011	San Souci	*O. borbonicus*	C			*	
RSB044	2012	Plan des Cafrès	*H. retusa*	C			*	
RSB054	2012	Plan des Cafrès	*H. retusa*	C			*	
RSB057	2012	Plan des Cafrès	*H. retusa*	C			*	
RSC046	2013	Palm des Palmiste	*O. borbonicus*	C			*	
RSC047	2013	Palm des Palmiste	*O. borbonicus*	C			*	
RSC048	2013	Palm des Palmiste	*O. borbonicus*	C			*	
RSC049	2013	Palm des Palmiste	*O. borbonicus*	C			*	
RSC050	2013	Palm des Palmiste	*O. borbonicus*	C			*	
RSC055	2013	San Souci	*H. retusa*	C			*	
RSC056	2013	San Souci	*H. retusa*	C			*	
RSC057	2013	San Souci	*H. retusa*	C			*	
RSC058	2013	San Souci	*H. retusa*	C			*	
RSC059	2013	San Souci	*H. retusa*	C			*	
RSC094	2013	Takamaka	*Adoretus* sp.	D			*	
RSC095	2013	Takamaka	*Adoretus* sp.	D			*	
RSC096	2013	Takamaka	*Adoretus* sp.	D			*	
RSC098	2013	Takamaka	*Adoretus* sp.	C			*	
RSC099	2013	Takamaka	*Adoretus* sp.	D			*	

STD, standard assays; TMP, temperature-based assays; MTP, multiple-choice assays; LIV, live-beetle assays.

### Chemosensation assays

#### Standard assays

Standard chemosensation assays largely followed the procedure outlined in Hong and Sommer (2006), which represents the standard procedure for free-living nematode chemoattraction studies (see Hong and Sommer 2006; and references therein). Briefly, a total of five organic compounds, four beetle wash extracts, and one pure pheromone extract were surveyed for their attractiveness to each of 21 *P. pacificus* strains (Table 1). These strains were selected because they are closely related genetically (all lineage “C” strains; see Morgan et al. 2012), thus largely removing genetic covariance effects from subsequent analyses. They were also chosen to cover a range of beetle hosts/locations (Table 1). The five organic compounds were purchased from Sigma–Aldrich (>97% purity; MO) and diluted in 100% ethanol (v/v) to varying concentrations (0.1%, 1%, 10% 100%; Table 2). These compounds/dilutions were selected based upon their ecological relevance (e.g., as insect allomone/pheromones or – because host finding can also involve indirect stimuli such as response to plant signals that are released upon attack by beetle larvae – as plant volatiles) and for comparison to the literature protocols (e.g., Hong and Sommer 2006). Further, the dilution range applied reflects the fact that true concentrations in nature remain unknown.

**Table 2:** The organic compounds tested against *Pristionchus pacificus* strains in chemoattraction assays, and their known source and function

Organic compound	Source	Function
Benzaldehyde	Fruit	Bitter almond extract in fruit
Ethyl myristate	Coleoptera/Diptera	Territorial marking pheromone
Methyl myristate	Coleoptera/Diptera	Allomone/pheromone
Toluene	Volcanoes	–
*Trans*-caryophyllene	Maize and other plants	Plant defense volatile
Beetle wash extracts		
*Adoretus* sp.	*Adoretus* sp. beetle specimens	Presumed allomone/pheromone
*Hoplia retusa*	*H. retusa* beetle specimens	Presumed allomone/pheromone
*Hoplochelus marginalis*	*H. marginalis* beetle specimens	Presumed allomone/pheromone
*Oryctes borbonicus*	*O. borbonicus* beetle specimens	Presumed allomone/pheromone
Pure pheromone extract		
Oryctalure	*Oryctes* species	Sex pheromone

For beetle wash extracts, adult beetles of four species collected from La Réunion in January 2010 and 2011 were soaked in dichloromethane for 24 h at room temperature or 23°C. The liquid was then transferred to a new tube, which was either air- or vacuum-dried at 30°C and resuspended in 120 *μ*L of pure ethanol. A solution of pure beetle pheromone extract was purchased from ChemTica Internacional, S.A. This extract was isolated from the *Oryctes* genus (Scarabaeidae, Coleoptera) and is commonly referred to as “Oryctalure.” Its formal designation and chemical formula are ethyl-(*RS*)-4-methyloctanoate and C_11_H_22_O_2_, respectively. It is commonly used in its pure (undiluted) form in agricultural applications as an insect attractant.

**Figure 2:**
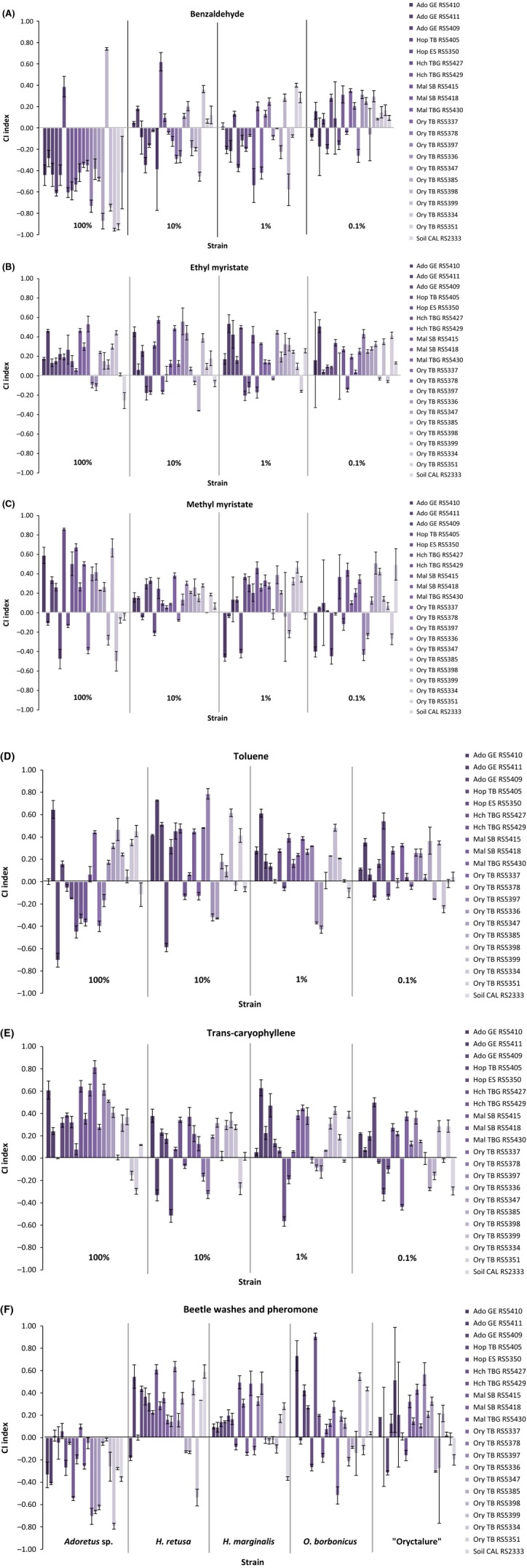
Natural variation in odor-response profiles of *Pristionchus pacificus* toward: (A) benzaldehyde; (B) ethyl myristate; (C) methyl myristate; (D) toluene; (E) *trans*-caryophyllene; and (F) beetle washes and the pheromone, “oryctalure.” Mean response ± standard deviation of 21 *P. pacificus* strains (including the control strain RS2333) to ten organic compounds (three replicates per assay) is shown. The chemotaxis index ranges from −1 to +1, with −1 indicating perfect repulsion and +1 indicating perfect attraction. Nematode origin information (original beetle host association and geographic location) is given in the key beside each figure for each strain (Ado, *Adoretus* sp.; Hop, *Hoplia retusa*; Hch, *Hoplochelus marginalis*; Mal, *Maladera affinis*; Ory, *Oryctes borbonicus*; GE, Grand Etang; TB, Trois Bassin; ES, Etang Salé; TBG, Trois Bassin Garden; SB, Saint Benoit; CAL, California, USA). All strains are of genetic lineage, “C,” except the reference strain, RS2333, which is lineage “A” (Morgan et al. 2012). See Tables 1, 3 for further information.

As per Hong and Sommer (2006), each standard chemosensation assay was performed on a plate of mixed-stage nematodes taken from 5-day-old cultures that were started with 10 hermaphrodites grown at 20°C. Before addition to the agar plate, nematodes were washed twice in PBS (+MgCl_2_) buffer and centrifuged briefly in 15-mL polypropylene tubes. A final concentrated suspension of nematodes was created by removing excess PBS buffer until approximately 100 nematodes were present per 100 *μ*L buffer (as determined over several trials by counting the number of nematodes in 100-*μ*L aliquots following sample preparation). On each plate, 1.5 *μ*L of attractant/counter-attractant and control (ethanol 100% or, in the beetle wash assays, dichloromethane) was spotted at opposite axes in addition to 1.5 *μ*L of 1 mol/L sodium azide, a mild anesthetic to prevent early attracted nematodes from adapting to the odor and leaving the area. Approximately 100 nematodes (see above) were added to the bottom of the plate at a point equidistant from the odor/control sources (Fig. 2A). Following a 15-h assay period (see Hong and Sommer 2006), plates were first placed briefly into a cold room (8°C) to immobilize nematodes, which were then scored for their location within a 0.5-cm radius circle of each odor source.

A chemotaxis index (CI) was calculated as a ratio for each assay: CI = [No. of animals at attractant−no. of animals at control]/[Total no. of nematodes at attractant and control] (see Hong and Sommer 2006). Final values for each assay were taken at the same time point (15 h), and the mean CI of all replicates was calculated. Three to five replicates of each assay containing at least ten scorable animals were carried out over the course of the experimental period for every condition tested. In all assays, we define compounds with a mean CI of ≥0.5 as attractive, 0.3–0.5 as weakly attractive, 0.29 to −0.29 as neutral, and ≤0.3 as repulsive (see Hong and Sommer 2006).

#### Temperature-based chemosensation assays

Given the variety of habitats across La Réunion Island, temperature is likely to be an important factor in environmental response among *P. pacificus* strains. To examine its potential impact on chemosensation variation, we performed a series of temperature-based chemosensation assays on a subset of six strains (Table 1). These strains were selected to encompass three beetle host/geographic location pairs and again were all lineage “C” strains (Morgan et al. 2012) to remove genetic covariance effects (Table 1). We tested these strains against three compounds: ethyl myristate (1%), *Oryctes borbonicus* beetle wash, and the pheromone extract, oryctalure (Table 2). All assay conditions replicated those outlined above, except, following addition of nematodes, assay plates were incubated at four different assay temperatures (15°, 20°, 25°, 30°C).

#### Multiple-choice assays

Standard chemoattraction assays (above) are capable of demonstrating a preference among strains for the potential attractant relevant to an ethanol control. To test whether strains could discriminate among one attractant relative to another, we performed a series of multiple-choice-based assays using beetle washes (Table 1). Here, the assay conditions replicated those of the standard assay, but five attractants were spotted evenly around the assay plate and nematodes were spotted in the center of the plate equidistant from these odor sources. The CI was then calculated as proportions among attractants relative to each other. A total of fifty assays were performed on strains that were selected to cover ten location/host beetle combinations (*n* = 5 per combination; Table 1), with each assay replicated three times, to determine whether nematodes would show relative chemoattraction preferences to beetle washes and whether any chemosensation result would relate to the original beetle species of nematode origin.

#### Live-beetle assays

Odor-guided behavior in the wild most likely occurs in response to blends of chemicals. We and others (e.g., Hong et al. 2008) have used beetle washes to overcome the limitation of chemosensation assays that are based on single compounds. In addition, a limited number of beetles collected from La Réunion Island in January 2011 were able to be returned alive to the Max Planck Institute for Developmental Biology (Tübingen, Germany) and maintained at 4°C. This provided an opportunity to develop a novel assay, using live beetles as the attractant source. Thus, several individuals of the beetles *Hoplochelus retusa* and *Hoplia marginalis* and a subset of nematode strains (Table 1) were selected in order to determine whether nematodes would chemotax to live beetles. Due to the limited number of live beetles available (*n* = 36 and 33 for *H. retusa* and *H. marginalis*, respectively), and the requirement to use three beetles per assay in *H. retusa* assays (since this beetle is comparatively small in size), this assay was only available for a select number of strains (four and 11 for *H. retusa* and *H. marginalis* assays, respectively). Hence, strains were chosen to cover a variety of beetle hosts and geographic locations (Table 1). These assays followed the standard protocol, with three replicates per assay, except beetles were held in a plastic vestibule that was inserted through the lid of an agar plate so that the tip of the vestibule hovered directly above one axis of the plate. This vestibule was open at the tip so that biological compounds from the live beetles could diffuse down onto the plate, which was sealed at all points of entry. The control in these assays consisted of an empty vestibule adhered to the opposite axis of the agar plate. All other conditions followed those employed for standard assays (above).

### Data analysis

All statistical analyses were performed in the programs Microsoft Office Excel ver. 2003 (Microsoft Corp., Redmond, WA) and Minitab ver. 14 (Minitab Inc., State College, PA).

The standard chemoattraction assays were analyzed using summary statistics (e.g., mean, standard deviation) to examine natural variation in chemotaxis index among strains across the various compounds and concentrations tested. An analysis of variance (ANOVA; general linear model, GLM) was used to detect whether response to compounds varied significantly among strains and to examine two potential factors (geographic location and host beetle species) for their effect on chemoattraction variation. Finally, cluster analysis was used to determine how similar the response of strains was toward the various organic compounds versus the beetle washes.

Analysis of variance (GLM) was also used to determine whether temperature accounted for variance in chemoattraction. In this analysis strain, temperature and their interaction were tested as factors.

Multiple-choice assays were analyzed using one-way ANOVA (with Tukey's *ad hoc* pairwise comparisons) to see if there were significant differences among strains in their response when presented with a choice of beetle washes. To examine whether any beetle wash preference in these assays was related to the original beetle host, strain location, or genetic lineage, ANOVA (GLM) was used.

Live-beetle assays were evaluated using summary statistics (mean, standard deviation) and one-way ANOVA to examine whether strains would chemotax to live beetles, and if so, whether the response among strains varied significantly.

## Results

### Chemosensation assays

#### Standard assays

Strong levels of natural variation underlie chemosensation in *P. pacificus*, with the chemoattraction response among strains differing significantly (*P* < 0.001) with tested compound (Table 3, Fig. 2).

**Table 3:** Results of analysis of variance (general linear model) test, examining the effects of compound, location, and beetle host on mean chemoattraction among 21 strains of *Pristionchus pacificus*. Note that location and beetle host had to be analyzed separately due to their colinearity

Source	df	Seq SS	Adj SS	Adj MS	*F*	*P*	S	R-Sq	R-Sq (adj)
Compound	24	13.081	13.081	0.545	7.01	<0.001	0.279	25.19%	21.60%
Error	500	38.855	38.855	0.077					
Total	524	51.937							
Location	5	0.549	0.549	0.110	1.11	0.354	0.315	1.06%	0.10%
Error	519	51.388	51.388	0.099					
Total	524	51.937							
Beetle host	5	0.410	0.410	0.082	0.83	0.531	0.315	0.79%	0.00%
Error	519	51.526	51.526	0.099					
Total	524	51.937							

df, degrees of freedom; SS, sums of squares; MS, means squares.

Among strains within compounds, a maximum range of mean variation of up to 1.69 CI units was seen – in response to benzaldehyde 100%, strain RS5398 (from *O. borbonicus* and Trois Bassin, TB; Table 1, Fig. 1) CI: 0.74 ± 0.01, RS5334 (*O. borbonicus*, TB): −0.95 ± 0.01 – representing nearly 85% of the total scale of the CI axis (Fig. 2A). Also in response to benzaldehyde 100%, two strains (RS5427 from Trois Bassin Garden, TBG and *H. marginalis*, and RS5398, above) showed attraction (0.39 ± 0.10 and 0.74 ± 0.01 for RS5427 and RS5398, respectively), but the majority of strains were repulsed (CI values ≤−0.30; Fig. 2A). Conversely, strain RS5411 (Grand Etang, GE, *Adoretus* sp.) was strongly attracted to toluene 100% (CI: 0.65 ± 0.08), while RS5409 (also from GE, *Adoretus* sp.) was strongly repulsed (CI: −0.70 ± 0.06; Fig. 2D).

Across concentrations of the same compound, strain responses also varied. For example, strain RS5427 (TBG, *H. marginalis*) showed both attractive and neutral responses to various concentrations of methyl myristate (0.86 ± 0.01, −0.21 ± 0.02, 0.37 ± 0.03, −0.01 ± 0.01, for 100%, 10%, 1%, and 0.1%, respectively; Fig. 2C).

Finally, across compounds, variation was detected (Fig. 2). Generally, most strains were repulsed by benzaldehyde and attracted to ethyl myristate, but strains showed a mix of attraction and repulsion responses to methyl myristate, toluene, and *trans*-caryophyllene. When presented with beetle washes, strains generally showed repulsion toward *Adoretus* sp. washes, and a medley of attractive and repulsive strain responses characterized the other washes and the oryctalure pheromone (Fig. 2F).

No dominant signal for preferential attraction among strains toward the beetle wash of the species they were originally isolated from was detected (Fig. 2F). For example, strains originally collected from *Adoretus* sp. generally showed repulsion or neutral responses to an *Adoretus* sp. beetle wash (−0.33 ± 0.12, −0.41 ± 0.01, 0.02 ± 0.05, for RS5410, RS5411, and RS5409, respectively). Conversely, strains collected from *H. retusa* were attracted to *H. retusa* beetle washes (0.44 ± 0.02 and 0.37 ± 0.08 for RS5405 and RS5350, respectively), while neutral responses characterized *H. marginalis*-collected strains (0.17 ± 0.06, −0.09 ± 0.03 for RS5427 and RS5429, respectively); responses toward *O. borbonicus*-collected strains ranged from repulsive (−0.52 ± 0.08 for RS5378) to attractive (0.55 ± 0.03 for RS5399) for the *O. borbonicus* beetle wash and from −0.30 ± 0.01 (RS5385) to 0.57 ± 0.10 (RS5397) for oryctalure (Fig. 2F).

**Figure 3:**
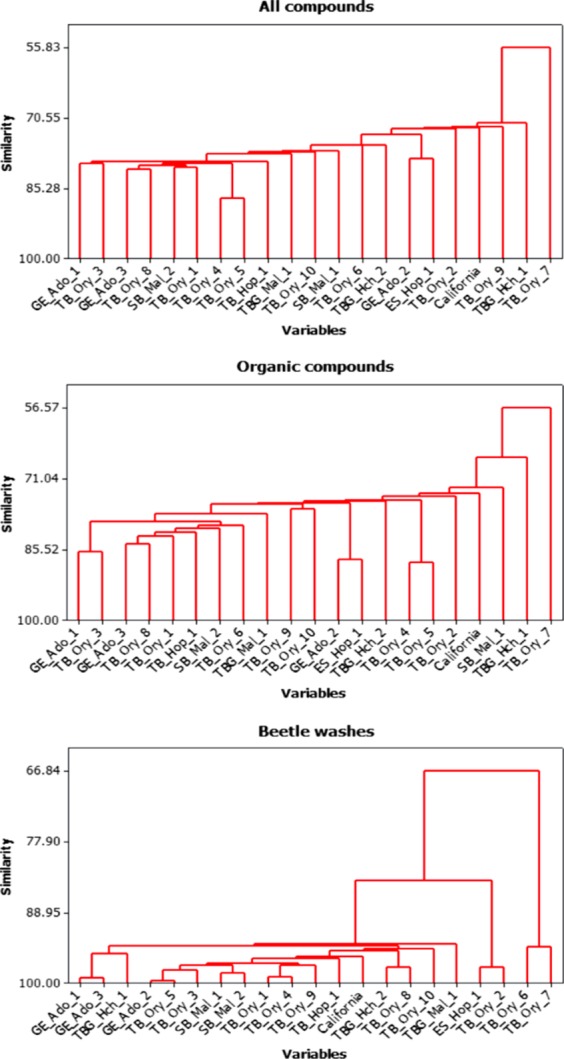
Results of cluster analysis, showing how similar the chemoattraction response of 21 *Pristionchus pacificus* strains was toward: all tested compounds, organic compounds only, and beetle washes only. Nematode origin information (original beetle host association and geographic location) is given on the *x-*axis for each strain (Ado, *Adoretus* sp.; Hop, *Hoplia retusa*; Hch, *Hoplochelus marginalis*; Mal, *Maladera affinis*; Ory, *Oryctes borbonicus*; GE, Grand Etang; TB, Trois Bassin; ES, Etang Salé; TBG, Trois Bassin Garden; SB, Saint Benoit; CAL, California; USA). All strains are of genetic lineage, “C,” except the reference strain, RS2333, which is lineage “A” (Morgan et al. 2012). See Tables 1, 4 for further information.

Analysis of variance (GLM) was subsequently used to formally test whether the original beetle host, as well as geographic location, significantly partitioned chemoattraction variance. In this analysis, neither factor significantly predicted CI variance (Table 3). This result was also seen in the cluster analysis, which showed that strain responses toward all compounds were quite dissimilar (Fig. 3A; Table 4). However, cluster analysis also showed that the response among strains was more similar in assays using beetle washes compared to organic compounds (Fig. 3B and C). For example, classifying the data into two clusters on the basis of strain CI values resulted in similarity/correlation coefficient distance levels among clusters of 84.037/0.319 for beetle wash assays, but 66.591/0.668 for organic compounds (Table 4). When we classified the data into 10 clusters, these values corresponded to 96.186/0.076 and 77.121/0.458 for beetle washes and organic compounds, respectively (Table 4); thus, similarity among strain responses was some 20% higher toward beetle washes than toward organic compounds (Fig. 3).

**Table 4:** Results of cluster analysis examining the similarity among 21 strains of *Pristionchus pacificus* in their chemoattraction responses toward: all compounds, organic compounds only, and beetle washes only

		All compounds			Organic compounds			Beetle washes		
				
Step	No. of clusters	S	D	No.	S	D	No.	S	D	No.
1	20	87.572	0.248	2	88.231	0.235	2	99.665	0.007	2
2	19	81.215	0.376	2	87.693	0.246	2	99.199	0.016	2
3	18	80.852	0.383	2	86.084	0.278	2	99.015	0.020	2
4	17	80.496	0.391	4	84.509	0.310	2	98.502	0.030	2
5	16	80.166	0.397	2	82.857	0.417	3	98.000	0.040	3
6	15	80.001	0.400	6	82.005	0.360	4	97.511	0.050	2
7	14	79.703	0.406	8	81.235	0.375	5	97.483	0.050	2
8	13	79.151	0.417	2	80.639	0.387	6	97.273	0.055	5
9	12	79.151	0.417	2	79.872	0.403	8	97.187	0.056	3
10	11	78.040	0.439	10	78.166	0.437	9	96.845	0.063	8
11	10	77.543	0.449	11	77.121	0.458	2	96.186	0.076	9
12	9	77.402	0.452	12	76.156	0.477	11	95.862	0.083	10
13	8	76.226	0.475	13	75.990	0.480	13	95.411	0.092	3
14	7	76.078	0.478	14	75.563	0.489	14	94.974	0.101	12
15	6	74.008	0.520	16	75.284	0.494	16	94.529	0.109	13
16	5	72.993	0.540	17	74.483	0.510	17	94.350	0.123	2
17	4	72.631	0.547	18	74.039	0.519	18	94.167	0.117	16
18	3	72.576	0.548	19	72.928	0.541	19	93.863	0.123	17
19	2	71.581	0.568	20	66.591	0.668	20	84.037	0.319	19
20	1	55.826	0.883	21	56.567	0.869	21	66.843	0.663	21

S, similarity level; D, correlation coefficient distance; No., number of observations in cluster.

#### Temperature-based chemosensation assays

**Figure 4:**
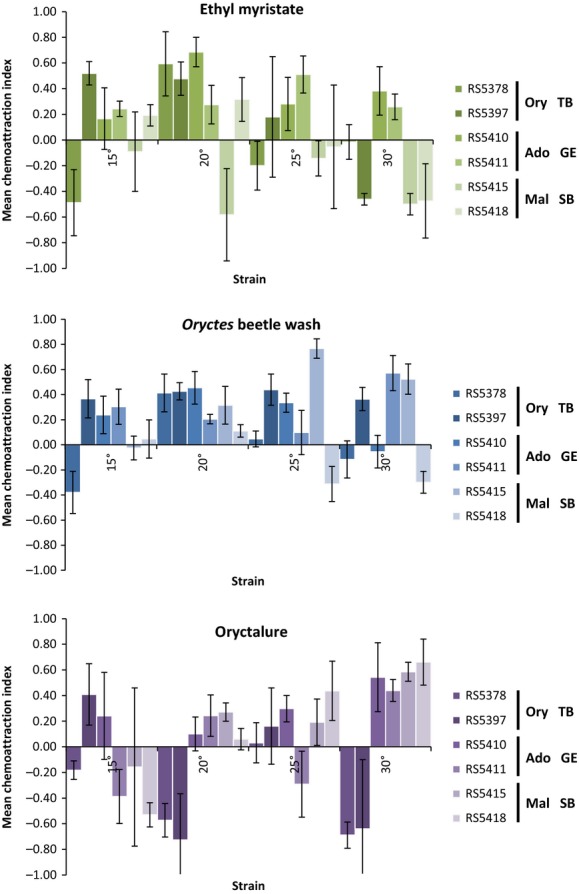
Results of the temperature-based chemoattraction assays for six *Pristionchus pacificus* strains. Results are presented for mean ± standard deviation chemoattraction to the compounds ethyl myristate (1%), *Oryctes borbonicus* beetle wash, and “Oryctalure” pheromone, at four measurement temperatures. Nematode origin information (original beetle host association and geographic location) is given to the right of the strain key for each isolate (Ory, *Oryctes borbonicus;* Ado, *Adoretus* sp.; Mal, *Maladera affinis*; TB, Trois Bassin; GE, Grand Etang; SB, Saint Benoit). All strains are of genetic lineage, “C” (Morgan et al. 2012). See Tables 1, 5 for further information.

The finding of natural variation at constant temperature (above) was reiterated in our temperature-based assays. In Figure 4, this is demonstrated by the large degree of scatter among sample responses generally, which varied within compounds, across temperatures, and across compounds. For example, response of strains from TB/*O. borbonicus* to ethyl myristate varied from −0.49 ± 0.26 to 0.52 ± 0.09 (RS5379 and RS5397, respectively), while response of RS5415 (Saint Benoit, SB; *Maladera affinis*) across compounds varied from −0.58 ± 0.36 (ethyl myristate) to 0.31 ± 0.15 (*O. borbonicus* beetle wash) and −0.73 ± 0.36 (oryctalure; Fig. 4). Response of a single strain across temperatures also varied. For example, strain RS5397 (TB/*O. borbonicus*) responses were 0.41 ± 0.24, −0.73 ± 0.36, 0.16 ± 0.30, and −0.64 ± 0.54 for 15°, 20°, 25°, and 30°C, respectively (Fig. 4). However, ANOVA (GLM) analysis found that differences in temperature among strains did not account for differences in chemoattraction response in a global analysis (Table 5). This analysis also found that the interaction between strain and temperature did not significantly partition CI variance (Table 5).

**Table 5:** Results of analysis of variance (general linear model) test, examining the effects of strain, temperature, and their interaction, on mean chemoattraction among six strains of *Pristionchus pacificus*

Source	df	Seq SS	Adj SS	Adj MS	*F*	*P*	S	R-Sq	R-Sq (adj)
Temperature	3	0.269	0.269	0.090	0.64	0.596	0.376	32.62%	0.33%
Strain	5	1.373	1.373	0.275	1.94	0.104			
Temperature*Strain	15	1.639	1.639	0.109	0.77	0.698			
Error	48	6.778	6.778	0.141					
Total	71	10.059							

df, degrees of freedom; SS, sum of squares; MS, means squares.

#### Multiple-choice assays

**Figure 5:**
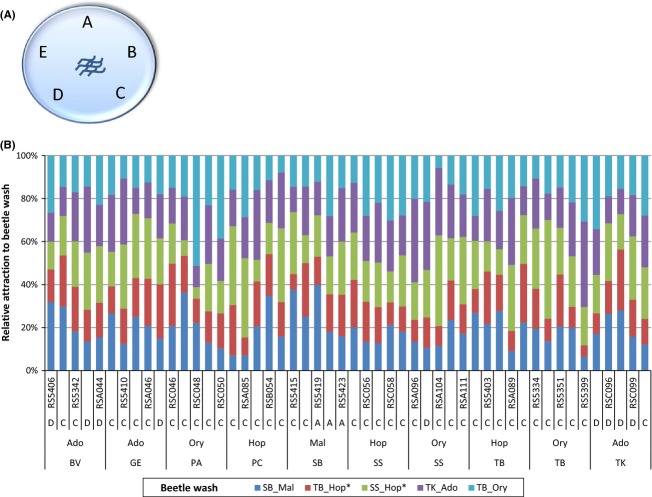
Results of multiple-choice assays: (A) Graphic to show the set-up of the assays, where five beetle wash attractants were spotted evenly around the assay plate at points A–E and nematodes were spotted in the center of the plate equidistant from these odor sources. The chemoattraction index (CI) was calculated as proportions among attractants relative to each other. A total of fifty assays using ten location/host beetle combinations (Table 1) were performed, with each assay replicated three times, to determine whether nematodes would show relative chemoattraction preferences to beetle washes; (B) Relative percentage of nematodes that chemotaxed to each point (A–E) on the assay plate for each assay for each beetle wash. Beetle wash is indicated by the key at the bottom of the figure (SB_Mal, Saint Benoit *Maladera affinis* beetle wash; TB_Hop, Trois Bassin *Hoplia retusa* beetle wash; SS_Hop, San Souci *H. retusa* beetle wash; TK_Ado, Takamaka *Adoretus* sp. beetle wash; TB_Ory, Trois Bassin *Oryctes borbonicus* beetle wash). Differences in attraction in response to the TB_Hop* and SS_Hop* washes were significant, as indicated by the “*” (Table 6). Nematode origin information (original beetle host association and geographic location) is given below the *x-*axis for each strain (Ado, *Adoretus* sp.; Ory, *Oryctes borbonicus;* Hop, *Hoplia retusa*; Mal, *Maladera affinis*; BV, Basse Vallée; GE, Grand Etang; PA, Palm des Palmiste; PC, Plan des Cafrès; SB, Saint Benoit; SS, San Souci; TB, Trois Bassin; TK, Takamaka). All strains are of genetic lineage, “A”, “C”, and “D” (Morgan et al. 2012), as indicated in Table 1.

When presented with a choice of five different beetle washes (Table 1), *P. pacificus* strains varied significantly in their chemoattraction response (*F*_4,245_ = 4.43; *P* = 0.002; Table 6). Tukey's pairwise analysis identified that these differences were due to mean strain responses to *H. retusa* beetle washes from TB and San Souci (SS), which were significantly different from both each other, and from 20% (the null proportion since five choices were presented; Fig. 5). Further analysis of the chemoattraction variance in a GLM framework showed that none of the analyzed factors (beetle host, genetic lineage, and location) significantly partitioned variance in CI for multiple-choice assays.

**Table 6:** Results of one-way analysis of variance (ANOVA) showing significant differences in the chemotaxis response among 50 *Pristionchus pacificus* strains toward a choice of beetle washes

Source	df	SS	MS	*F*	*P*	S	R-Sq	R-Sq (adj)
ANOVA								
Factor	4	0.102	0.025	4.43	0.002	0.076	6.74%	5.22%
Error	245	1.409	0.006					
Total	249	1.510						

Factor	*N*	Mean	Standard deviation
Individual 95% confidence intervals for mean			
SB_Mal	50	0.196	0.081
TB_Hop	50	0.165	0.058
SS_Hop	50	0.224	0.084
TK_Ado	50	0.215	0.070
TB_Ory	50	0.199	0.084

df, degrees of freedom; SS, sum of squares; SS_Hop, sums of squares *H. retusa* beetle wash; MS, means squares; TK_Ado, Takamaka *Adoretus* sp. beetle wash; TB_Ory, Trois Bassin *Oryctes borbonicus* beetle wash.

#### Live-beetle assays

**Figure 6:**
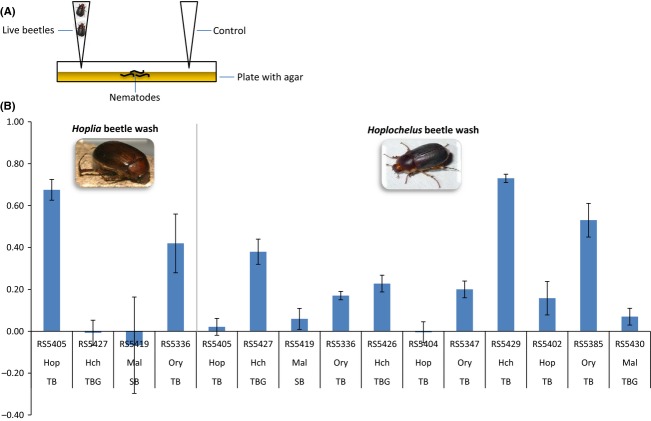
Results of live-beetle assays for *Pristionchus pacificus*; (A) Schematic of the experimental set-up; (B) Variation in odor-response profile of selected *P. pacificus* strains in live-beetle assays using the beetles *Hoplia retusa* (left four strains) and *Hoplochelus marginalis* (right 11 strains) from La Réunion Island. Results are presented as follows: mean chemotaxis index ± standard deviation. Nematode origin information (original beetle host association and geographic location) is given below the *x-*axis for each strain (Hop, *Hoplia retusa*; Hch, *Hoplochelus marginalis*; Mal, *Maladera affinis*; Ory, *Oryctes borbonicus*; TB, Trois Bassin; TBG, Trois Bassin Garden; SB, Saint Benoit). All strains are of genetic lineage, “A” or “C” (Morgan et al. 2012). See Table 1 for further information.

In live-beetle assays, strains showed they were able to chemotax toward the signal produced by live beetles. In responding to the *H. retusa* live beetle, strain response (*n* = 4) varied from neutral (−0.07 ± 0.23 for RS5419 from SB/*M. affinis*) to strongly attracted (0.68 ± 0.05 for RS5405 from TB/*H. retusa*. Response to the *H. marginalis* live beetle showed similar variation among the 11 strains tested: 0.00 ± 0.05 for RS5404 from TB/*H. retusa* to 0.73 ± 0.02 for RS5429 from TB/*H. marginalis* (Fig. 6). One-way ANOVA analysis found these differences in attraction to live beetles to be significant among strains (*F*_14,30_ = 27.23, *P* < 0.001, *r*^2^ = 92.70% for all strains; *F*_3,8_ = 19.04, *P* < 0.001, *r*^2^ = 87.72% for strains responding to *H. retusa* beetle wash; *F*_10,22_ = 59.43, *P* < 0.001, *r*^2^ = 96.43% for strains responding to *H. marginalis* beetle wash; Fig. 6).

## Discussion

An understanding of organism–environment interactions requires, as a first step, characterization of the potential traits involved in an organisms' sensation of its surroundings. Here, we show that one trait that is particularly important for nematode response to environmental stimuli, chemosensation, exhibits strong levels of variation among strains. This variation was present in *P. pacificus* across all of our assays, with strains varying in response in a given assay over a range covering up to 85% of the possible chemosensation index.

As well as strains showing different responses to a given compound, variation was present across concentrations of the same compound and across compounds themselves. Cluster analysis of the standard assay data showed that responses were indeed dissimilar among strains, and further statistical analysis indicated that temperature, beetle host, and sampling location were not responsible for chemoattraction variance. However, cluster analysis also showed that responses among strains were more similar when the test compound was a beetle wash than when it was an organic compound. Given that these nematodes show a more concerted response toward the compounds they most likely directly encounter in the wild, this suggests that variation in the odor-guided response of *P. pacificus* may have an important ecological component.

Our mix of standard, temperature-based, multiple-choice and live-beetle assays showed that *P. pacificus* is able to discriminate between test compounds. For example, in standard assays, strains showed a general pattern of repulsion toward benzaldehyde and attraction to ethyl myristate. Further differences existed in comparisons of response of organic versus beetle wash assays, and our live-beetle assays demonstrated that strains are capable of responding to volatile cues released by a live beetle. Our multiple-choice assays also indicated that different nematode strains could show relative chemoattraction preferences to beetle washes. The ability of *P. pacificus* to discriminate among various chemical cues in the current study further supports the ecological importance of this trait. A related nematode, *Pristionchus maupasi*, can use such discrimination to specifically recognize older feeding beetles, thereby reducing its dauer period to the shorter-lived adult host stage (Hong et al. 2008). Whether *P. pacificus* is also capable of discrimination across beetle developmental stages is an interesting consideration for future work.

Discrimination across beetle species in the form of preferential host choices could have several important effects on the population dynamics of *P. pacificus*. For example, on La Réunion Island, distinct beetles are constrained by different diversities, distributions, and dispersal capacities (Morgan et al. 2012). Thus, host choices will likely result in exposure to different local environmental conditions encountered by the beetle. Alternatively, if choice of beetle host affects the amount/duration of bacterial food resources available, or their efficiency as feeding sources for *P. pacificus* (see Rae et al. 2008), then, on certain beetle host species, a greater number of generations could occur before nematodes must arrest development, thus possibly influencing strain and/or population survival. Given these potential host choice effects, the lack of a dominant signal for preferential attraction among strains to their original beetle host in our multiple-choice assays is somewhat surprising. However, cluster analysis did show a concerted response among strains toward beetle washes compared to organic compounds, and it may be that our dataset lacked the power (*n* = 5 strains per host/location combination and *n* = 3 replicates per assay) to detect a clear preference pattern. Alternatively, strains may behave differently under laboratory conditions or have become acclimated after several generations of selfing without host beetle contact.

The variability of chemoattraction in *P. pacificus* suggests that response to different volatile cues in this free-living nematode, aside from showing similarity among beetle washes, may be more of a strain-specific characteristic than one influenced by location or temperature. Strain-specific responses independent of temperature have also been found previously in both entomopathogenic and parasitic nematodes (Ali et al. 2010, 2011), suggesting that plasticity both within and among populations and/or hosts may be an important feature of the chemoattractive response for some nematodes. Conversely, chemosensation in other species has been shown to be an isolating force among populations. For example, chemosensation has been shown to lead to premating isolation among populations and species of moth (Smadja and Butlin 2009; and references therein) and to behavioral isolation in flies of the genus *Drosophila* (Etges and Ahrens 2001; Ferveur 2005; Smadja and Butlin 2009). In *C. elegans*, phenotypic variation in chemosensation has been suggested to be closely related to fitness (Schulenburg and Müller 2004; Barrière and Fèlix 2005) and therefore potentially under selection.

In *P. pacificus*, a large degree of variability within both individuals and populations characterizes chemosensation, as shown here and in Hong et al. (2008), but also a number of other traits (e.g., Bento et al. 2010; Mayer and Sommer 2011). Across species, much experimental data suggest that individual differences in behavioral strategies employed at the intrapopulation level are important in optimizing fitness (e.g., Ehlinger 1990; Paffenhöfer 1994; Utne et al. 1997; Sneddon 2003; Van Oers et al. 2005). Thus, the interindividual variability identified here may suggest that chemosensation variation plays a role in population persistence for *P. pacificus* by allowing strains to better match their phenotype to their environment (Viney and Diaz 2012).

We have demonstrated a substantial degree of natural variation in chemosensation for *P. pacificus* and highlighted the ability of this nematode to discriminate among a variety of chemical cues. We suggest that divergence in odor-guided behavior in *P. pacificus* may have an important ecological component, whereby strains show a concerted response toward the compounds they most likely directly encounter in the wild. Our results provide a comprehensive basis for the evolution and ecology of *P. pacificus* chemosensation. It would be useful to investigate whether other ecological traits of the nematodes of La Réunion are diverging, and future work should focus on elucidation of the genetic basis and evolutionary mechanisms underlying such trait divergence (e.g., Hong et al. 2008; Bento et al. 2010; Mayer and Sommer 2011). With such studies, the future of this system promises success in bridging the boundaries of ecological context, phenotypic expression, gene function, and molecular variation.
